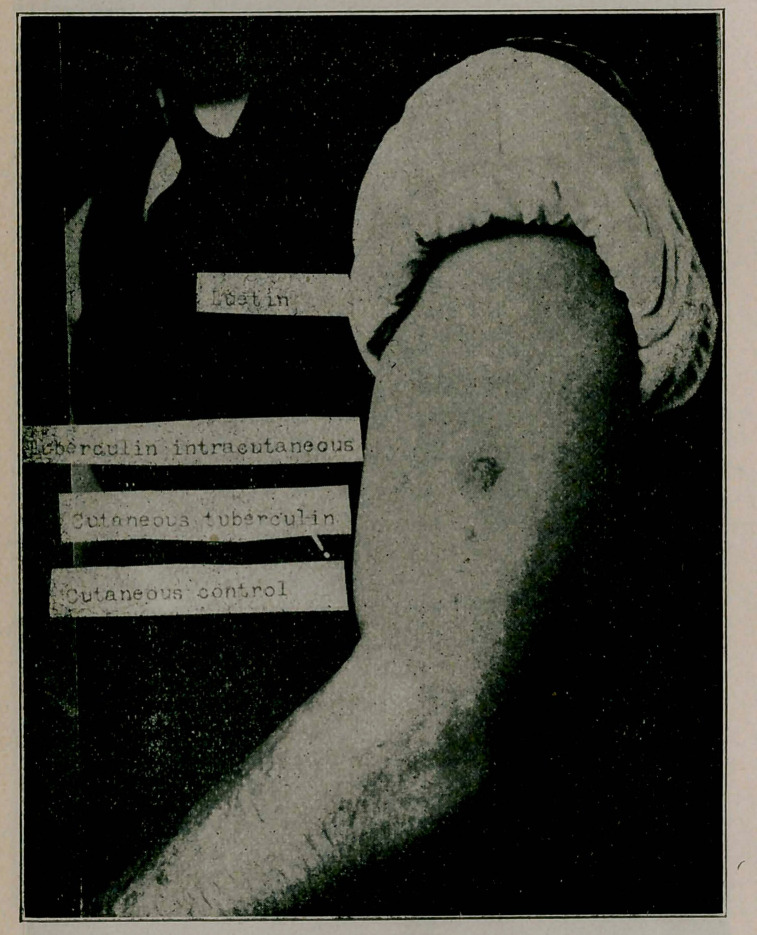# The Effect of Certain Drugs upon Skin Reactions

**Published:** 1918-05

**Authors:** 


**The Effect of Certain Drugs Upon Skin Reactions.** John A. Kolmer, Samuel L. Immerman, Toitsu Matsunami, and

Charles M. Montgomery. The Journal of Laboratory and Clinical Medicine, Mch. 1917. Illustrate the various confusions of reactions with luetin and tuberculin tests and report laboratory studies of the strengths necessary to cause phagocytic and other reactions. (Cuts by courtesy of authors and publisher). They summarize as follows:

1. The iodides and particularly potassium iodide were found to influence the luetin and prodigiosis intracutaneous tests to a marked extent. Normal non-syphilitic persons, reacting negatively in the luetin test, may show marked reactions when tested after the oral administration of sixty or more grains of potassium iodide.

2. The bromides of potassium and sodium in the same dosage were found to have a similar but less marked influence.

3. The chlorides of potassium and ammonium in the same dosage were found to influence the prodigiosin reactions but not the luetin reactions except to a very slight extent.

4. The administration of the protiodide of mercury influenced the luetin reaction to some extent.

5. It is probable that the administration of larger doses of these drugs would exert a more marked influence upon skin reactions.

6. Ether and chloroform anesthesia did not appear to influence skin reactions.

7. Substances most likely to excite inflammatory reactions in the skin appear to be most readily influenced by the iodides and to some extent by bromides; intracutaneous tests with agar-agar, prodigiosin and ordinary luetin were more readily influenced by these drugs than the reactions following the injection of a luetin of washed spirochetes and free of culture medium.

8. Cutaneous tests are not as readily influenced by these drugs as intracutaneous tests.

9. Conjunctival tests among normal rabbits to tuberculin were apparently not influenced.

10. Cutaneous and intracutaneous reactions to tuberculin among persons reacting positively to both, appear to be rendered more extensive by potassium iodide and to a lesser extent by potassium bromide.

11. Anaphylactic reactions to luetin in syphilitic persons appear to be rendered more extensive by potassium iodide and potassium bromide.

12. The oral administration of potassium iodide and to a lesser extent of potassium bromide, increased the phagocytic power of the blood serum for B. prodigiosis; the increased severity of skin reactions in persons taking these drugs may be due to heightened leucocytic infiltration and phagocytosis about the injected material or to an increase of tryptic activity through the saturation of fatty acid radicals according to the hypothesis of Jobling and Petersen.

13. Physicians should very carefully rule out the possible influence of these drugs before conducting skin reactions.

14. It is probable that these drugs have influenced the luetin reactions as clinically applied and have been responsible in part for the divergent results observed and reported.

## Figures and Tables

**Figure f1:**
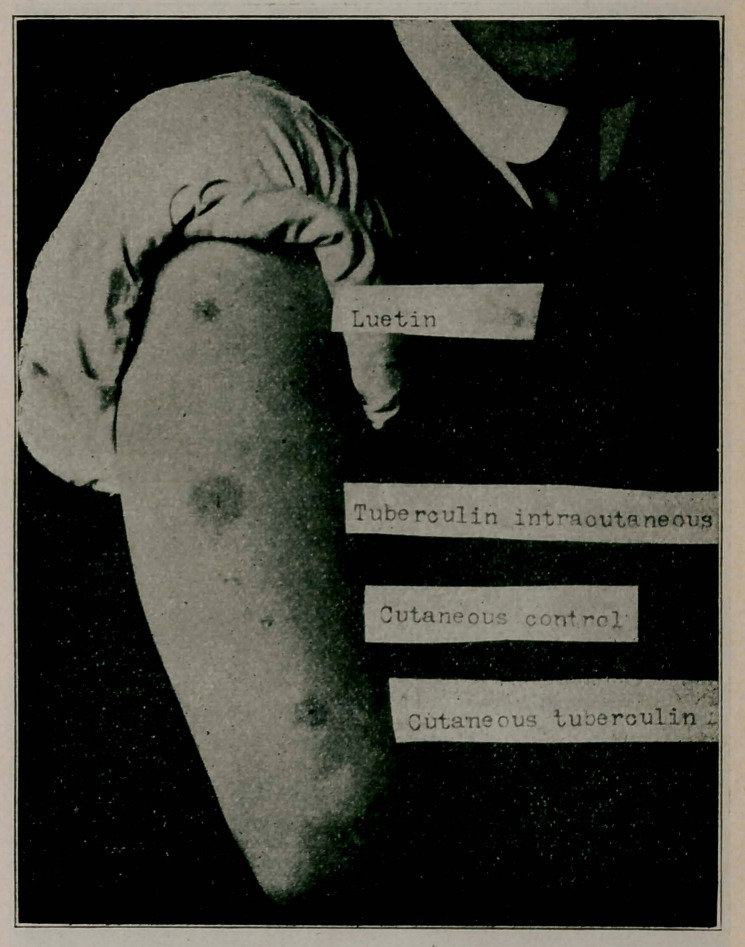


**Figure f2:**
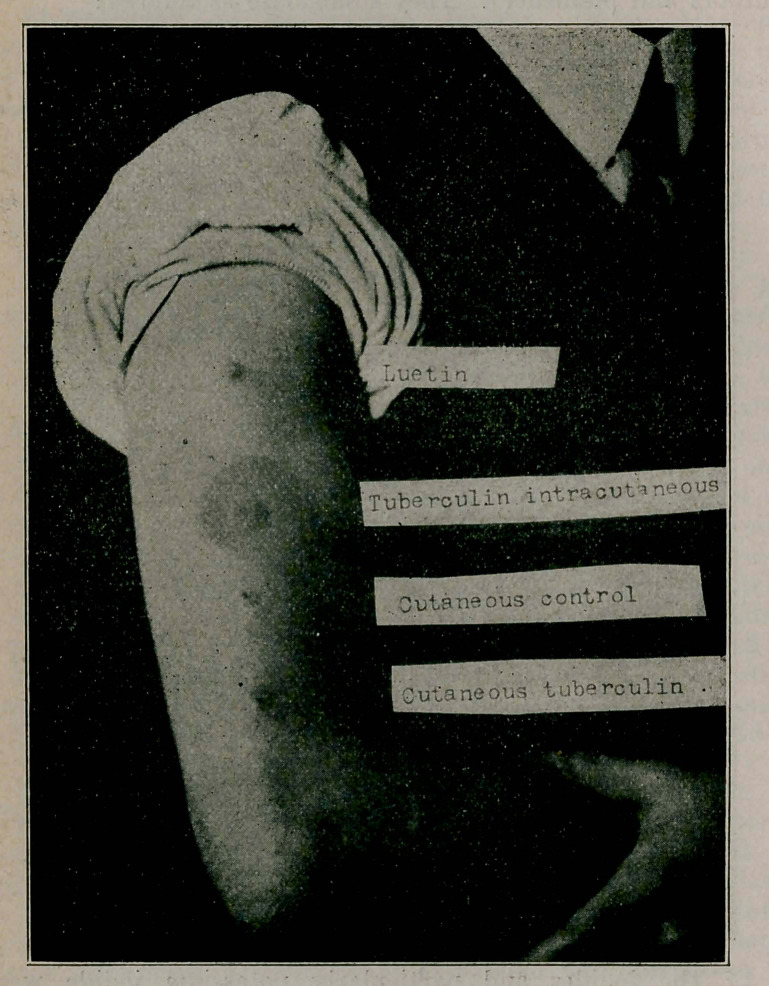


**Figure f3:**
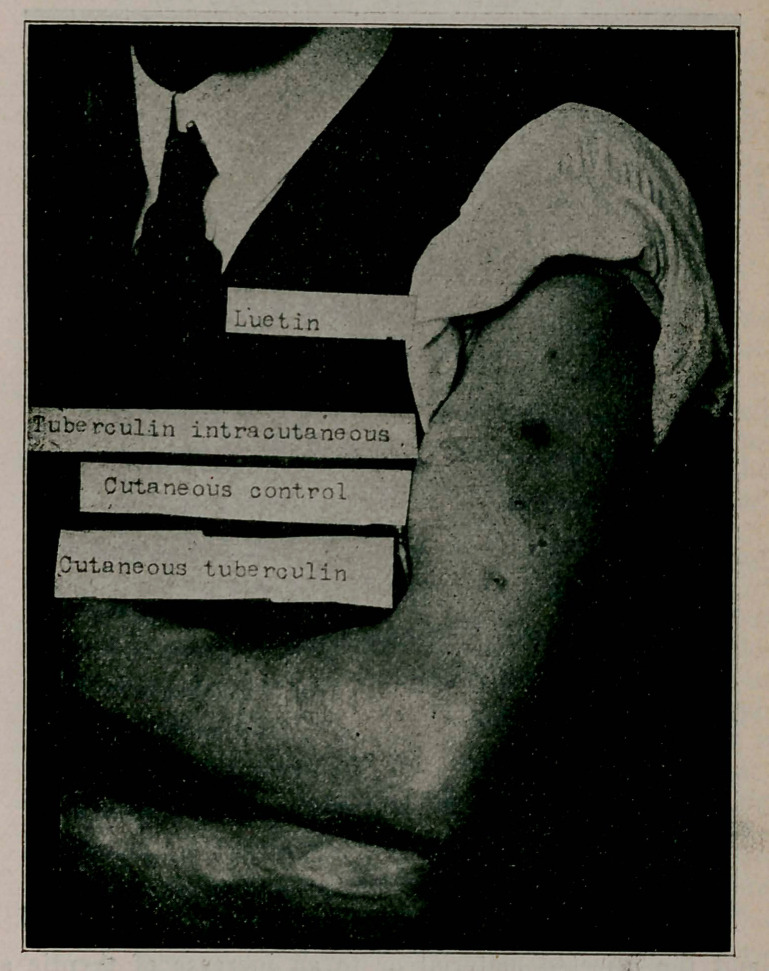


**Figure f4:**